# High fat diet increases melanoma cell growth in the bone marrow by inducing osteopontin and interleukin 6

**DOI:** 10.18632/oncotarget.8474

**Published:** 2016-03-30

**Authors:** Guang-Liang Chen, Yubin Luo, Daniel Eriksson, Xianyi Meng, Cheng Qian, Tobias Bäuerle, Xiao-Xiang Chen, Georg Schett, Aline Bozec

**Affiliations:** ^1^ Department of Internal Medicine 3, University of Erlangen-Nuremberg, Erlangen, Germany; ^2^ Minhang District Central Hospital, Fudan University, Shanghai, China; ^3^ Department of Thoracic Surgery, Zhongshan Hospital, Fudan University, Shanghai, China; ^4^ Department of Rheumatology, Renji Hospital Affiliated to Shanghai Jiao Tong University School of Medicine, Shanghai, China; ^5^ Institute of Radiology, University Medical Center Erlangen, Friedrich-Alexander University Erlangen-Nuremberg, Erlangen, Germany

**Keywords:** bone tumor microenvironment, obesity, melanoma, bone marrow adipocyte, osteopontin

## Abstract

The impact of metabolic stress induced by obesity on the bone marrow melanoma niche is largely unknown. Here we employed diet induced obese mice model, where mice received high-fat (HFD) or normal diet (ND) for 6 weeks before challenge with B16F10 melanoma cells. Tumor size, bone loss and osteoclasts numbers were assessed histologically in the tibial bones. For defining the molecular pathway, osteopontin knock-out mice, interleukin 6 neutralizing antibody or Janus kinase 2 inhibition were carried out in the same model. Mechanistic studies such as adipocyte-melanoma co-cultures for defining adipocyte induced changes of tumor cell proliferation and expression profiles were also performed. As results, HFD enhanced melanoma burden in bone by increasing tumor area and osteoclast numbers. This process was associated with higher numbers of bone marrow adipocytes expressing IL-6 in direct vicinity to tumor cells. Inhibition of IL-6 or of downstream JAK2 blocked HFD-induced tumor progression. Furthermore, the phenotypic changes of melanoma cells triggered macrophage and osteoclast accumulation accompanied by increased osteopontin expression. Osteopontin triggered osteoclastogenesis and also exerted a positive feedback loop to tumor cells, which was abrogated in its absence. Metabolic stress by HFD promotes melanoma growth in the bone marrow by an increase in bone marrow adipocytes and IL-6-JAK2-osteopontin mediated activation of tumor cells and osteoclast differentiation.

## INTRODUCTION

Around 39% of the world adult population is overweight, and 13% obese. Epidemiological, clinical and experimental studies have shown that over nutrition leading to adipose tissue expansion increases the risk of malignant melanoma (MM) and worsens its prognosis [1–8]. Bone metastases derived from melanoma are very common (23-49%) in autopsy series but also frequently observed in clinical series (5%-17%) [9]. After diagnosis of bone metastases, the patient's life expectancy is rather short with a median survival up to 2-6 months [9]. Although better therapies are currently available for the treatment of metastatic melanoma [10–13], the prognosis of patients with bone metastasis is still poor [13, 14]. To improve the prognosis of metastatic melanoma, interventions could be required, which target both the disseminating seed and the metastatic soil (or niche) [11, 15].

The bone marrow hematopoietic stem cell (HSC) niche is key for cancer cells to engraft into bone [15–18]. During obesity, the HSC niche is largely disturbed by the increased presence of adipocytes in the bone marrow. It is surprisingly unknown whether obesity-induced bone marrow changes affect the homing and survival of melanoma cells in their respective niches. Obesity is characterized by increase in adipocyte numbers and size. In adipocyte-rich soft tissue such as the breast, adipocytes interact dynamically with cancer cells and induce cancer cell growth, invasion and metastasis [19–21]. Moreover, following contact with cancer cells, mature adipocytes undergo dedifferentiation or apoptosis, which in turn activates pro-inflammatory cytokine secretion by macrophages within the tumor microenvironment [22–25]. Dedifferentiation of adipocytes usually leads to interleukin (IL) -6 production, which is important for tumor invasiveness [23, 25] and macrophage differentiation [24, 26].

Adipocytes, however, are not only present in subcutaneous fat tissue but also found in large quantities in the bone marrow (BM), where mesenchymal stem cells differentiate into the bone forming osteoblasts or into adipocytes. Balance between bone marrow osteoblasts and adipocytes assures the maintenance of the bone mass and the integrity of the bone marrow space. Indeed, BM adipocyte content increase with obesity [27]. As bone marrow adipocytes are essential for HSC maintenance and myeloid cell differentiation [28], they may exert an important role in building tumor cell niches. In support of this concept, one previous study mentioned that BM adipocytes modulate the fatty acid binding protein 4 (FABP4)-interleukin-1 beta (IL-1β) pathway in prostate cancer cells, when homing into bone [29]. Furthermore, adipocytes, in particular their interaction with macrophages has been considered important for increased lymph node metastasis of melanoma cells during diet-induced obesity [21]. On the other hand, MM can induce adipocyte apoptosis and thereby activate macrophages through osteopontin [30], an important cytokine for osteoclast differentiation [31, 32].

In the present study, we defined the role of obesity on the homing and survival of melanoma cells in the bone marrow niches. We demonstrate that diet-induced obesity fosters the activation of tumor-associated macrophages and osteoclasts by bone marrow adipocytes via IL-6. We further delineate that expression of osteopontin, IL-6 and the melanoma derived chemokine C-X-C motif ligand 1 (CXCL1) in the bone marrow increases after high fat diet, predisposing melanoma cells to home into the bone likely due to enhanced osteoclast activation. Most convincingly, blockade of IL-6 or osteopontin rescued obesity-induced melanoma growth into the bone marrow and normalized osteoclast activation.

## RESULTS

### High fat diet increases melanoma growth in the bone and increases bone-resorbing osteoclasts

**Figure 1:**
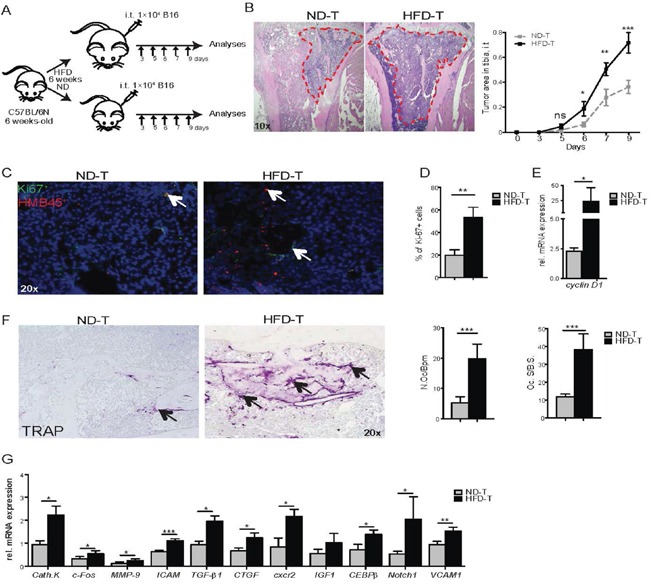
High fat diet mice have an increased bone tumor growth correlated with tumor-infiltrating osteoclasts/macrophages **A.** Experimental scheme: mice fed for 6 weeks with normal diet (ND) or high fat diet (HFD) were injected intratibially (i.t.) with B16F10 cells (1×10^4^) in PBS (50 μl) or with vehicle (PBS, 50 μl). Then, mice were sacrificed at day 3, 5, 6, 7, and 9 post tumor inoculation. **B.** Hematoxilin & Eosin (HE) stained pictures of tibiae from ND and HFD mice at day 7 post i.t. B16F10 cell injection (magnification ×10). Tumor areas are shown by red dotted line. Quantification of the tumor growth at the indicated time point. **C-D.** Ki67 staining (C) and Ki67^+^ cells quantification (D) in bone tumor area from ND and HFD mice at day 7 post i.t. B16F10 cell injection (magnification ×20). Arrows indicate Ki67^+^ cells. **E.** *Cyclin D1* mRNA levels in bone from ND and HFD mice at 7 days post i.t. B16F10 cells injection. **F.** TRAP staining pictures in bone tumor area from ND or HFD mice (magnification ×20). Histomorphometric osteoclast quantification in the tumor center of ND or HFD mice. Abbreviations: N.Oc/B.Pm, Number of osteoclasts per bone perimeter; Oc.S/BS, osteoclast surface/bone surface. **G.** Osteoclast and macrophage gene markers expression in bone from ND and HFD mice 7 days post i.t. B16F10 cells injection. All data are means ± SEM; n=6 to 8 per group. *p<0.05, **p<0.01, ***p<0.001.

To determine whether high fat diet (HFD)-induced obesity impacts melanoma growth in bone, 6 week-old C57BL/6N male mice were fed for 6 weeks with HFD (60 kcal% fat) or normal diet (ND, 10 kcal% fat). Next, HFD and ND-treated mice were injected with B16F10 melanoma cells into the tibial bone marrow (Figure 1A). In the intratibial tumor model, bone tumor volume was significantly higher in HFD compared to ND mice already 6 days after tumor cell injection (Figure 1B). When characterizing tumor cell proliferation, we found an increased number of Ki67 positive cells, consistent with increased *Cyclin D1* mRNA levels in tumor cells of HFD compared to ND mice (Figures 1C-1E).

To determine whether the bone was affected, osteoclasts were quantified. Osteoclast numbers were significantly higher in the tumor microenvironment of HFD mice compared to ND-treated mice (Figure 1F). In contrast, no difference in osteoclast numbers between ND versus HFD treated mice were observed in non-injected mice (data not shown), despite a decreased bone volume in non-injected or tumor cell injected HFD mice when compared to ND (Figure S1). Molecular profiling for osteoclasts and macrophage markers revealed increased expression of *cathepsin K, c-Fos, matrix metalloproteinase 9 (MMP-9), intercellular adhesion molecule 1 (ICAM1), transforming growth factor β1 (TGFβ1), connective tissue growth factor (CTGF), chemokine (C-X-C motif) receptor 2 (CXCR2), CCAAT/enhancer-binding protein beta (CEBPβ), notch homolog 1, translocation-associated (Notch1)* and *vascular cell adhesion molecule 1* (*VCAM1)* in HFD- compared to ND-treated mice 7 days after tumor cell challenge (Figure 1G). All together, these data showed increased tumor burden in bone as well as enhanced osteoclast numbers after exposure to HFD.

### High fat diet increases melanoma cell proliferation and osteoclastogenesis

**Figure 2:**
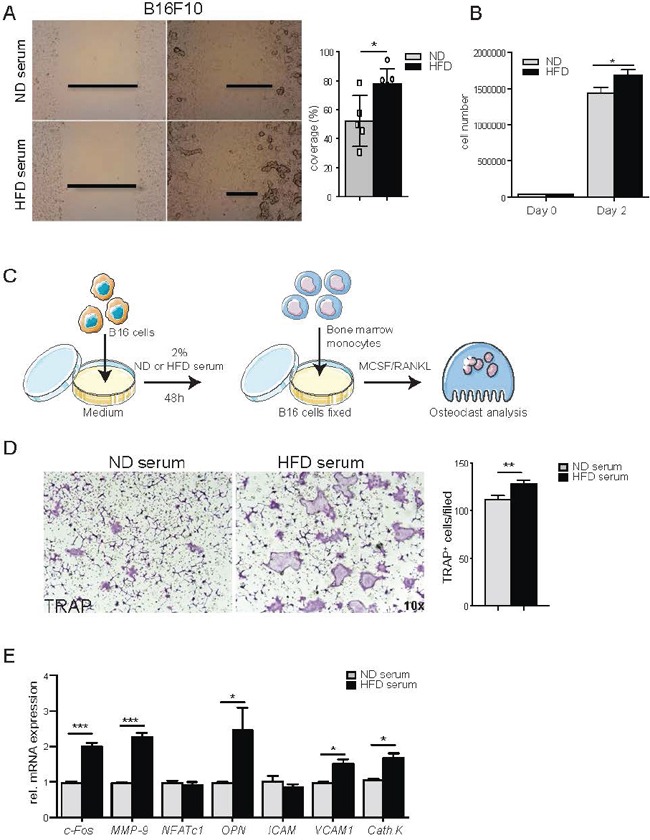
Systemic factors from high fat diet serum induce tumor cell proliferation, migration and osteoclast differentiation **A.** Scratch assay of B16F10 cell treated with 2% normal diet (ND) or high fat diet (HFD) serum. A representative image of at least 3 independent experiments carried out in triplicate was shown. Percentage of B16F10 cell coverage after 8 hours of scratch. **B.** Quantification of B16F10 cells number after treatment with 2% ND or HFD serum at the indicated time point. **C.** Scheme overview of the *in vitro* experiments: B16F10 cells (5×10^4^) are coated on 24-well plate and stimulated with 2% serum from ND or HFD mice. After 12h treatment, B16F10 cells are fixed and co-cultured with BM derived monocytes in presence of M-CSF and RANKL to induce osteoclast (Oc) differentiation. **D.** Representative picture of TRAP staining of Oc cultures in presence of B16F10 cells pre-treated with ND or HFD serum (magnification 10x). TRAP positive osteoclasts (nuclei ≥3) are counted. **E.** Gene expression of osteoclast markers in osteoclast/B16F10 co-culture cells. All data are means ± SEM; 3 independent experiments were carried out in triplicate. *p<0.05, **p<0.01, ***p<0.001.

To determine whether circulating factors present in high fat diet (HFD) mice could influence melanoma cell proliferation *in vitro*, we treated B16F10 cells with serum derived from ND or HFD mice. As shown in Figures 2A-2B and Figure S2A-S2B, migration and proliferation rate of melanoma cells were higher after HFD than ND serum exposure. The S or G2M phases were decreased after HFD serum treatment (Figure S2B). Gene expression analyses of proliferation, adhesion and angiogenesis markers showed significant increase of expression levels of *cyclin D1 (CCND1), v-akt murine thymoma viral oncogene homologue 1 (Akt1), mitogen-activated protein kinase 3 (MapK), forkhead box a1 (Foxa1), ICAM1, cadherin 2 (N-cad), C-X-C chemokine receptor type 7 (CXCR7), vascular endothelial growth factor-C (VEGF-C), transforming growth factor-β2 (TGFβ2) and angiopoietin-2 (Ang2)* in tumor cells treated with HFD-derived serum (Figure S2C), while no difference was observed for the other parameters. Taken together these results show that HFD enhances melanoma cell growth *in vitro* and *in vivo*.

Next, we tested whether melanoma cells exposed to HFD serum affect osteoclastogenesis. Indeed, quantification of TRAP+ cells resembling bone-resorbing osteoclasts showed that melanoma cells exposed to HFD-serum significantly enhanced osteoclast differentiation (Figures 2C-2E, Figure S3A-S3C). However, conditioned medium from melanoma cells pre-treated with HFD or ND serum was not sufficient to stimulate osteoclast differentiation (Figure S3D-S3F). Taking together, these findings indicated that melanoma cells activated by HFD enhance osteoclast differentiation.

### Metabolic stress by high fat diet increases osteopontin level

**Figure 3:**
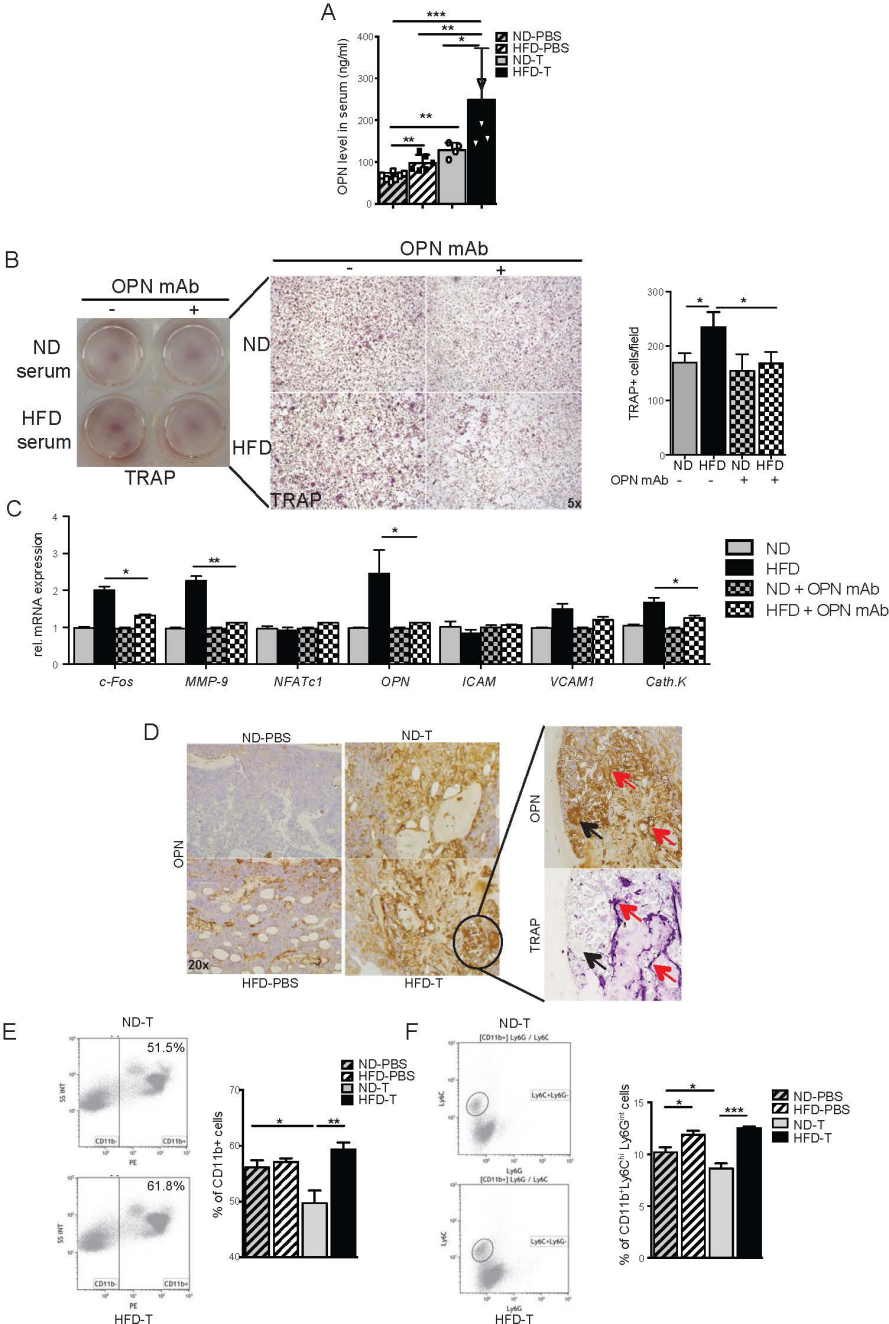
Osteopontin is essential for the osteoclast induction and is produced by CD11b+ monocytes in bone marrow from high fat diet mice **A.** Serum levels of osteopontin (OPN) in normal diet (ND) and high fat diet (HFD) naive mice or intratibially (i.t.) injected with B16F10 cells. **B.** B16F10 cells were pre-treated with 2% ND or HFD serum or OPN depleted serum for 12h, and co-cultured with BM derived monocytes. TRAP staining pictures and TRAP^+^ osteoclasts quantification after 6 days of induction with MCSF and RANKL (magnification 10x). **C.** Real time analyses of osteoclast markers in cell cultures. **D.** OPN staining in tibia from ND and HFD mice at day 7 post i.t. B16F10 cells injection and TRAP staining in serial slide of the HFD B16F10 injected tibia (magnification ×20). Red arrows indicate co-localization staining and black arrow show no co-localization staining. **E-F.** Representative flow cytometry plots and quantification of CD11b^+^ (E) and CD11b^+^Ly6C^hi^Ly6G^int^ (F) populations present in tibia of ND and HFD mice 7 days post i.t. B16F10 injection. All data are means ± SEM; (*in vitro*) 3 independent experiments were carried out in triplicate. (*in vivo*) n=6 to 8 per group. *p<0.05, **p<0.01, ***p<0.001.

Since obesity is known to induce inflammation [33, 34], we hypothesized that increased cytokine levels in HFD serum could be responsible for melanoma cell activation. Therefore, cytokines were analyzed by FACS array in the sera of mice treated with either ND and HFD and challenged with melanoma cells. Out of the 13 cytokines tested (Table 2), osteopontin level was significantly higher in the sera of HFD compared to ND mice and further increased after melanoma cell challenge (Figure 3A). Therefore, we addressed the role of osteopontin in the crosstalk between melanoma cells and osteoclasts. As shown in Figure 3B, the stimulatory effect of HFD serum on osteoclast was abolished when osteopontin was blocked (Figure 3B). Similarly, HFD- induced elevation of osteoclast markers normalized after depletion of osteopontin (Figure 3C). Collectively, these data suggested that the increased osteoclast activation in tumor area results from the increase of osteopontin brought on by HFD.

**Table 1:** Primer sequence list

Gene name	Forward sequence (5′ to 3′)	Reverse sequence (5′ to 3′)
*Adiponectin*	GCG ATA CAT ATA AGC GGC TTC T	GCA GGC ATC CCA GGA CAT C
*Akt1*	GGACTACTTGCACTCCGAGAAG	CATAGTGGCACCGTCCTTGATC
*Ang2*	CTCTGTCTCAGGATGACTCCAG	AGGTGTTGACATCTTTGCAGAAAG
*aP2*	TGAAATCACCGCAGACGACAGG	GCTTGTCACCATCTCGTTTTCTC
*β-Actin*	TGTCCACCTTCCAGCAGATGT	AGCTCAGTAACAGTCCGCCTAGA
*Cathepsin K*	AGGGCCAACTCAAGAAGAAAACT	TGCCATAGCCCACCACCAACACT
*CCND1*	GCAGAAGGAGATTGTGCCATCC	AGGAAGCGGTCCAGGTAGTTCA
*CD44*	CGGAACCACAGCCTCCTTTCAA	TGCCATCCGTTCTGAAACCACG
*CD74*	GCTGGATGAAGCAGTGGCTCTT	GATGTGGCTGACTTCTTCCTGG
*CEBPα*	AAGAGCCGCGACAAGGC	GTCAGCTCCAGCACCTTGTG
*CEBPβ*	CAACCTGGAGACGCAGCACAAG	GCTTGAACAAGTTCCGCAGGGT
*c-Fos*	CGG GTT TCA ACG CCG ACT AC	CAG GTC TGG GCT GGT GGA GA
*c-Myc*	TCGCTGCTGTCCTCCGAGTCC	GGTTTGCCTCTTCTCCACAGAC
*CTGF*	CTGTCAAGTTTGAGCTTTCTGG	GGACTCAAAGATGTCATTGTCC
*CXCL1*	TCCAGAGCTTGAAGGTGTTGCC	AACCAAGGGAGCTTCAGGGTCA
*CXCL14*	TACCCACACTGCGAGGAGAAGA	CGCTTCTCGTTCCAGGCATTGT
*CXCL2*	CATCCAGAGCTTGAGTGTGACG	GGCTTCAGGGTCAAGGCAAACT
*CXCL5*	CCGCTGGCATTTCTGTTGCTGT	CAGGGATCACCTCCAAATTAGCG
*CXCR2*	CTCTATTCTGCCAGATGCTGTCC	ACAAGGCTCAGCAGAGTCACCA
*CXCR4*	GTGTAAGGCTGTCCATATCATC	GACAGCTTAGAGATGATGATGC
*CXCR7*	GACCGCTATCTCTCCATCACCT	GTTGGAAGCAGATGTGACCGTC
*Foxa1*	GCCTTACTCCTACATCTCGCTC	CTGCTGGTTCTGGCGGTAATAG
*HPRT*	GCTTGCTGGTGAAAAGGACCTC	CAAATCAAAGTCTGGGGACGC
*ICAM1*	AAACCAGACCCTGGAACTGCAC	GCCTGGCATTTCAGAGTCTGCT
*IGF1*	GTGGATGCTCTTCAGTTCGTGTG	TCCAGTCTCCTCAGATCACAGC
*IL-1β*	TGGACCTTCCAGGATGAGGACA	GTTCATCTCGGAGCCTGTAGTG
*IL-6*	TCCTTCCTACCCCAATTTCC	GCCACTCCTTCTGTGACTCC
*Jag-1*	AACGACCGTAATCGCATCGT	TATCAGGTTGAATAGTGTCATTACTGGAA
*Krt18*	AATCAGGGACGCTGAGACCACA	GCTCCATCTGTGCCTTGTATCG
*Leptin*	ATCTGAAGCAAGCCATCAGC	CCAGTCACCAGAGGTCAAGC
*M-cadherin*	AGGACGAGCATAGCTGAAGGAG	GTCCACTTGCAGCCAGTCTTCT
*MCSF*	GCCTCCTGTTCTACAAGTGGAAG	ACTGGCAGTTCCACCTGTCTGT
*MMP9*	GCTGACTACGATAAGGACGGCA	TAGTGGTGCAGGCAGAGTAGGA
*Mpa3K*	GGCTTTCTGACGGAGTATGTGG	GTTGGAGAGCATCTCAGCCAGA
*N-cadherin*	TCGCTGCTTTCATACTGAACTTT	AGCGCAGTCTTACCGAAGG
*NFATc1*	GGTGCCTTTTGCGAGCAGTATC	CGTATGGACCAGAATGTGACGG
*Notch1*	GCTGCCTCTTTGATGGCTTCGA	CACATTCGGCACTGTTACAGCC
*Nrp1*	TAC CTC ACA TCT CCC GGT TAC C	GAA GAT TTC ATA GCG GAT GG
*OPN*	TCCTTAGACTCACCGCTCTT	TCTCCTTGCGCCACAGAATG
*PDGFα*	CTGGCTCGAAGTCAGATCCACA	GACTTGTCTCCAAGGCATCCTC
*PI3K*	CAAACCACCCAAGCCCACTACT	CCATCAGCAGTGTCTCGGAGTT
*PIGF*	AGTTTCACAGGAGCGTGGCTTG	GATCCAGAGTGGCGAGATAACC
*PPARγ2*	CTGATGCACTGCCTATGAGC	GGGTCAGCTCTTGTGAATGG
*Pref1*	GACACTCGAAGCTCACCTGG	GGAAGGCTGGGACGGGAAAT
*Pten*	TGAGTTCCCTCAGCCATTGCCT	GAGGTTTCCTCTGGTCCTGGTA
*S100a8*	CAAGGAAATCACCATGCCCTCTA	ACCATCGCAAGGAACTCCTCGA
*Sema3a*	GACATCTATGGCAAAGCCTGTGC	GTGAGTCAGTGGGTCTCCATTC
*TGFβ1*	TGATACGCCTGAGTGGCTGTCT	CACAAGAGCAGTGAGCGCTGAA
*TGFβ3*	AAGCAGCGCTACATAGGTGGCA	GGCTGAAAGGTGTGACATGGAC
*VCAM1*	GCTATGAGGATGGAAGACTCTGG	ACTTGTGCAGCCACCTGAGATC
*VEGFα*	CTGCTGTAACGATGAAGCCCTG	GCTGTAGGAAGCTCATCTCTCC
*VEGFc*	CCTGAATCCTGGGAAATGTGCC	CGATTCGCACACGGTCTTCTGT
*VEGFR2*	CATCACCGAGAACAAGAACA	CATTGATCTTTGCCTCACAG

**Table 2:** Cytokine multiplex analysis of serum from naive and B16 injected mice

Cytokine (pg/ml)	ND-PBS (n=4)	HFD-PBS (n=4)	ND-T (n=6)	HFD-T (n=6)	*P* value
IL-10	16.64±0.6502	15.7 ±0.4788	16.81±0.8853	16.4±1.129	0.3259
IL-6	23.11±0.4793	23.06±0.2533	25.01±0.9555	75.46±16.79	0.0017
IL-1α	30.04±5.599	27.56±2.248	27.57±1.522	52.32±52.79	0.5318
IL-2	19.11±1.531	19.2±1.053	18.78±1.457	18.5±1.951	0.8959
IL-5	41.52±2.258	40.91±4.24	41.77±1.387	44.04±8.007	0.7912
GM-CSF	9.048±0.5649	9.15±0.25	9.209±0.1484	9.224±0.2568	0.8562
IFNγ	43.33±2.106	44.23±1.398	47.85±7.667	46.31±6.85	0.6525
IL-17	89.17±1.743	91.17±2.603	90.19±0.5007	103.2±11.08	0.0167
IL-4	26.81±0.9498	27.97±0.6894	27.52±0.5861	27.19±0.5928	0.1590
TNFα	55.02±2.837	56.11±2.028	55.36±1.349	60.8±14.86	0.7086

All data are means ± SEM. One-way analysis of variance.

### Increase in osteopontin-producing macrophages in the bone marrow tumor area

To address, which cells express osteopontin, bone marrow of HFD and ND treated mice having been challenged with melanoma cells was stained with anti-osteopontin antibody. Osteopontin was expressed by macrophages and osteoclasts in the bone marrow (Figure 3D). Also, TRAP-negative multinucleated cells around the tumor expressed osteopontin (Figure 3D). When further analyzing the macrophage population in the bone marrow by FACS, we found higher percentages of CD11b+ cells and CD11b+Ly6C+Ly6G^int^ cells in HFD compared to ND mice (Figures 3E-3F). These data suggest an accumulation of osteopontin- producing macrophages in the bone marrow of HFD treated mice.

### B16F10 cells become more “aggressive” in the presence of adipocytes

**Figure 4:**
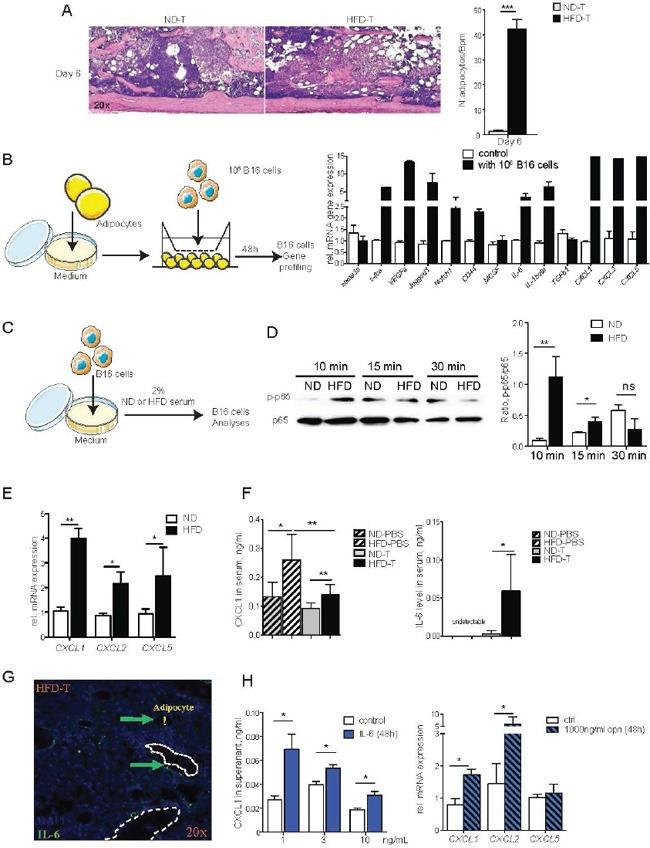
The NF-κB and CXCLs pathways in B16F10 cells are induced by high fat diet serum, IL-6 and OPN **A.** Hematoxilin & eosin stained tibial sections showing the bone marrow tumor and adipocytes in mice treated with normal diet (ND) and high fat diet (HFD). Mice received B16F10 tumor cells 6 days before the analysis. Bar graph shows quantification of adipocyte numbers in the bone marrow. **B.** Transwell co-culture with B16F10 melanoma cells and bone marrow adipocytes: Gene profiling of melanoma cells after 48 hours of co-culture with adipocytes. **C.** Experimental outline: B16F10 cells were treated with 2% serum from ND or HFD mice. **D.** Western blotting analysis of phospho-NF-κB p65 (Ser536) and NF-κB p65 level in B16F10 cells after treatment. The intensive ratio is quantified by Image-J software. **E.** mRNA levels of *CXCL1, CXCL2* and *CXCL5* in B16F10 cells treated with ND or HFD serum. **F.** Serum levels of CXCL1 and IL-6 in ND or HFD post i.t. injection with B16F10 cells. **G.** Immunofluorescence staining of IL-6 in bone tumor microenvironment from HFD mice. Arrows indicate IL-6 positive cells. Exclamation mark shows bone marrow adipocyte. **H.** CXCL1 levels in supernatant of BM derived macrophage treated with IL-6. *CXCL1*, and *CXCL2* mRNA levels in bone marrow derived macrophages treated with OPN. All data are means ± SEM; (*in vitro*) 3 independent experiments were carried out in triplicate. (*in vivo*) n=6 to 8 per group. *p<0.05, **p<0.01, ***p<0.001.

To better understand the increase in bone marrow macrophages in mice challenged with HFD and tumor cells, we considered that an interaction between fat and tumor cells may happen, which promotes a local inflammatory response. Therefore, we first examined whether HFD triggers an enhance anatomical connection between tumor cells and adipocytes. Indeed, bone marrow adipocytes were significantly more abundant in the bone marrow of HFD treated mice and were directly associated to the site of tumor growth (Figure 4A). To address whether adipocytes influence the function of melanoma cells, we performed adipocyte/melanoma cell co-cultures, which showed an adipocyte triggered increase of mediator expression by the melanoma cell. The factors included the pro-inflammatory cytokines IL-6 and IL-1β as well as the chemokines CXCL1, CXCL2 and CXCL5 (Figure 4D). Furthermore, exposure of melanoma cells to HFD serum induced the pro-inflammatory NF-κB pathway (Figures 4B-4C) and also enhanced the expression of *CXCL1, CXCL2* and *CXCL5* (Figure 4E). In accordance, IL-6 and CXCL1 serum levels increased after HFD exposure *in vivo*, similar to serum levels of osteopontin (Figure 4F). Importantly, both IL-6 and osteopontin up-regulated chemokine mRNA levels in a time course dependent manner in the melanoma cells (Figure 4H). All together, these data indicate that adipocyte/melanoma cell cross-talk induces pro-inflammatory cytokines and chemo-attractant secretion, which likely mediates the infiltration of macrophages in the bone marrow.

### Enhanced tumor growth in obese mice is mediated by IL-6-JAK2-osteopontin axis

**Figure 5:**
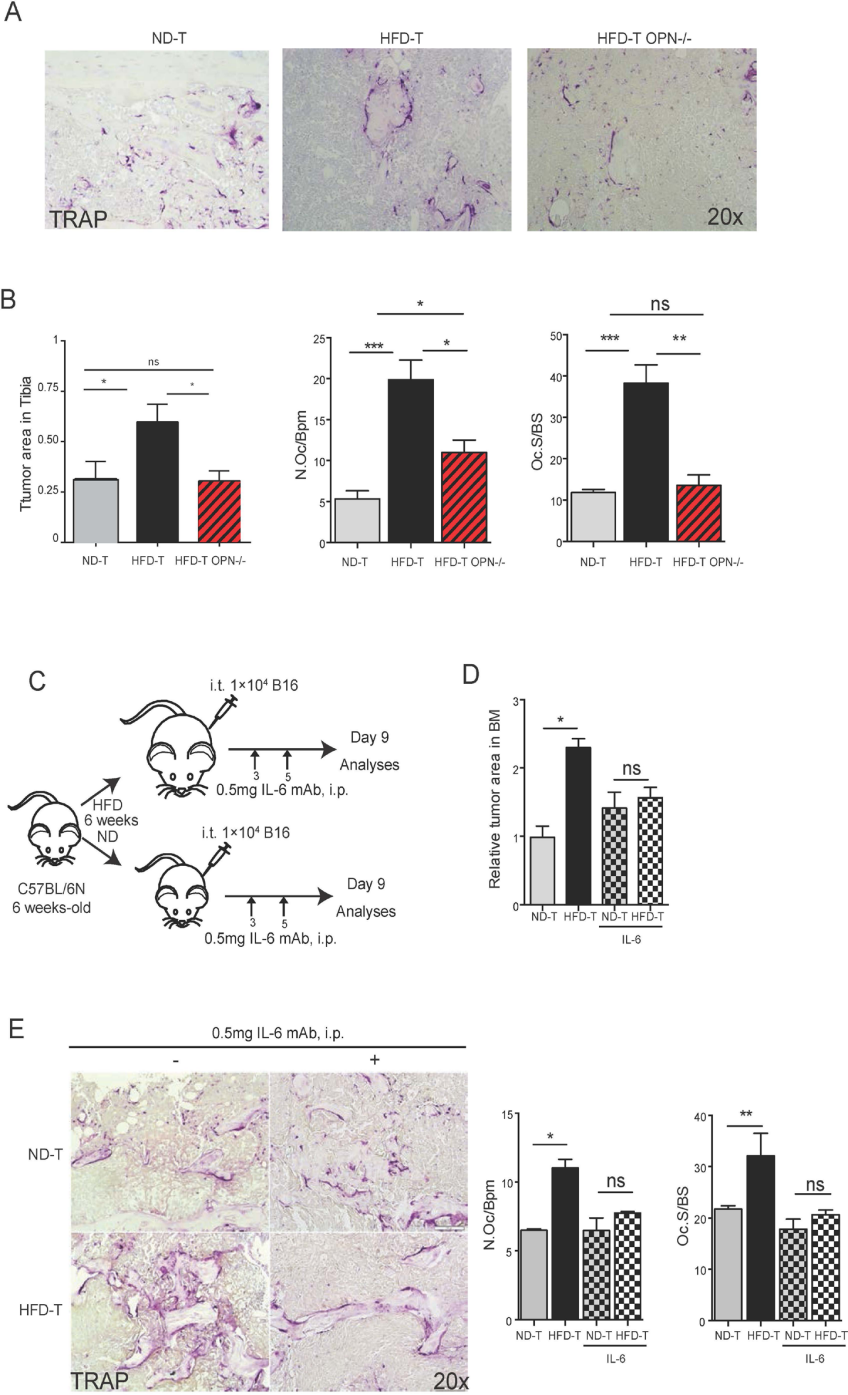
OPN deficiency or mAb IL-6 treatments rescue the increased tumor growth in tibia of high fat diet mice **A.** Representative pictures of TRAP staining in normal diet (ND), high fat diet (HFD) wild-type or HFD deficient OPN mice at day 7 post intratibial (i.t.) B16F10 cells injection (magnification x20). **B.** Quantification of tumor growth, osteoclast number and surface in tibia from ND, HFD wild-type or HFD deficient OPN mice at day 7 post i.t. B16F10 cells injection. **C.** Schematic pictures for experimental setting of IL-6 mAb (0.5 mg/mouse) injection. **D.** Relative tumor area in ND or HFD mice injected with B16F10 cells with or without mAb IL-6 treatment. **E.** Representative pictures of TRAP staining (magnification x20) and quantification of osteoclast number and surface in ND or HFD mice injected with B16F10 cells with or without mAb IL-6 treatment. All data are means ± SEM; (*in vitro*) 3 independent experiments were carried out in triplicate. (*in vivo*) n=4 to 8 per group. *p<0.05, **p<0.01, ***p<0.001.

**Figure 6:**
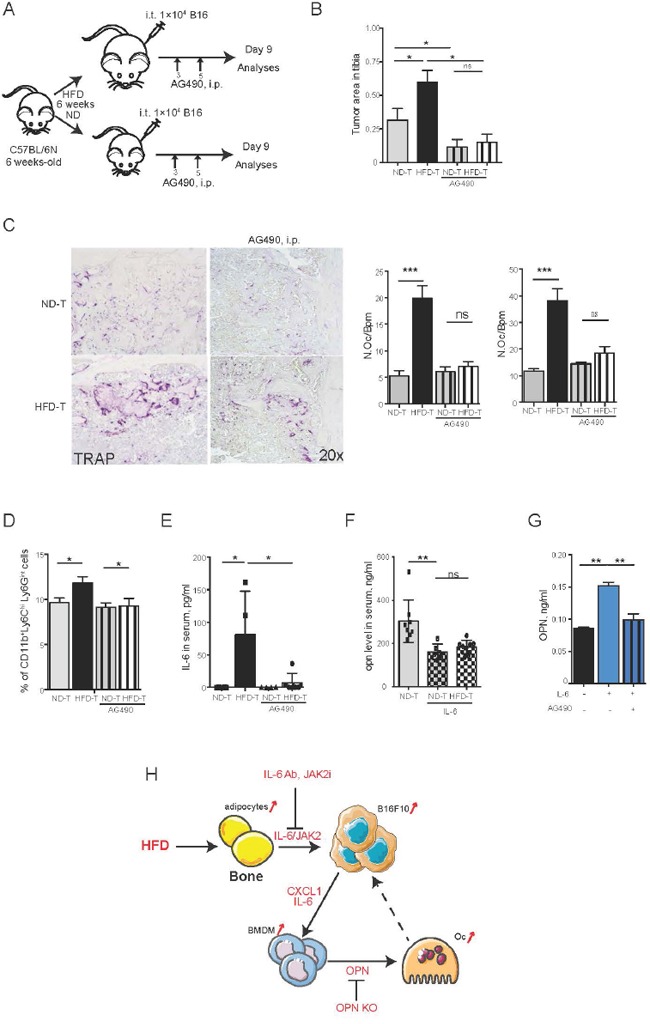
Blockade of JAK2-STAT3 pathway rescues the tumor size and the number of osteoclasts in high fat diet mice **A.** Schematic picture of the experimental setting: AG490 is intraperitoneally injected every day till day 9 with the dose of 17 mg/kg/mouse. **B.** Quantification of tumor size in tibia of each group. **C.** Representative pictures of TRAP staining and osteoclast number and size in each group. **D.** FACS analysis of CD11b^+^Ly6C^hi^Ly6G^int^ populations in bone marrow from tumor bearing mice. **E-F.** IL-6 (E) and OPN (F) levels in serum of normal diet (ND) or high fat diet (HFD) mice injected with B16F10 cells with or without AG490 treatment. **G.** OPN levels in supernatant from bone marrow derived macrophages treated with IL-6 recombinant and AG490. **H.** A model of crosstalk between tumor cells, adipocytes and macrophages in HFD tumor microenvironment. All data are means ± SEM; (*in vitro*) 3 independent experiments were carried out in triplicate. (*in vivo*) n=6 to 8 per group. *p<0.05, **p<0.01, ***p<0.001.

To further determine the role of osteopontin and IL-6 for the bone marrow tumor niche, we used osteopontin deficient (OPN-/−) mice and neutralizing IL-6 monoclonal antibody treatment, respectively. Interestingly, OPN-/− mice showed significantly reduced tumor burden and significantly lower osteoclast numbers, when challenged with HFD, compared to wild-type controls (Figures 5A-5B), suggesting that osteopontin was a tumor- promoting and osteoclastogenic factor during HFD. Furthermore, neutralization of IL-6 had a very similar effect, showing virtual rescue of enhanced tumor growth and osteoclast activation by HFD (Figures 5C-5E). To further analyze the role of IL-6, we blocked downstream JAK2 by specific inhibitor AG490. As shown in Figures 6A-6C, AG490 injection reduced tumor growth as well as osteoclast activation in HFD-treated mice. These data indicated that osteopontin and IL-6 are responsible for the enhanced tumor growth in obese mice.

### Osteopontin is induced by IL-6 in bone marrow macrophages

Consistent with the rescue of tumor growth and osteoclast activation by JAK2 inhibitor, also the HFD-induced elevation of bone marrow CD11b+Ly6C+Ly6G^int^ cells and systemic IL-6 levels were abolished (Figures 6D-6E and data not shown). Moreover, serum osteopontin level was also decreased after blocking JAK2 (Figure 6F), which was consistent with IL-6 mediated up-regulation of osteopontin expression in BM-derived macrophages (Figure 6G). Similarly, the *in vivo* data inhibition of JAK2 by AG490 effectively blocked osteopontin secretion by BM-derived macrophages (Figure 6G). These data demonstrated that IL-6 and osteopontin are the main mediators for enhanced melanoma growth in bones following HFD metabolic stress.

## DISCUSSION

Here, we show that metabolic stress by HFD, which is associated with an increase in adipose tissue, enhances melanoma burden. Melanoma is one of the most aggressive cancers, which disseminate and metastasize to multiple sites including bone [35]. These findings raised the question about the mechanism, how adipose tissue influences tumor growth a phenomenon which is increasingly recognized in the tumor field [4, 6, 8, 21, 36] and becomes important facing a pandemics of obesity in developed countries. Melanoma cells homing to the bone marrow are directly exposed to bone marrow fat, which may influence tumor growth and provide a suitable tumor microenvironment. Mechanistically, the numbers of tumor-associated adipocytes and macrophages significantly increased after HFD, which triggered tumor cells growth. Molecularly, activation of the IL6-JAK2-OPN axis was essential for mediating enhanced melanoma growth during HFD.

We have recently shown that adipocytes numbers increase in the bone marrow during HFD, which changes the hematopoietic stem cell pool in the bone marrow microenvironment, and results in a shift from lymphoid to myeloid cell differentiation [28]. We speculated that these bone marrow changes provide an ideal micro-environment for tumor growth providing melanoma cells a differentiation niche. Indeed, our data support the concept that adipocytes in the bone marrow drive melanoma growth. Our results are also supported by previous observations showing adipocyte number increased in the bone marrow with age [37, 38] and the prevalence of bone metastases in elderly melanoma patients [39–42]. Despite several reports on IGF1, Rankl, leptin and the cytokine IL-8, the molecular signaling linking bone marrow adipocyte and bone tumor growth remains to be fully elucidated [37, 12, 38, 19, 23]. Here we showed that adipocytes in the vicinity of the tumor cells express large amounts of IL-6, which can promote tumor growth. Similar observations have also been made in breast cancer models [22]. Furthermore, earlier data have shown that adipocyte numbers in the bone marrow affect leptin levels, which were shown to accelerate melanoma growth [6]. However, leptin levels decreased during tumor progression in HFD mice (data not shown), suggesting that it may not be involved in the high tumor burden in the bone of HFD-exposed mice. In support of the important role of IL-6 in promoting cancer growth in the bone marrow, blockade of IL-6 by neutralizing antibody as well as inhibition of JAK2 by small-molecule inhibitors significantly blocked tumor growth. This finding is important since inhibitors of IL-6, such as tocilizumab and JAK2, such as ruxolitinib, are already in clinical use for the treatment of rheumatoid arthritis and osteomyelofibrosis, respectively.

Activation of melanoma cells by HFD includes induction of the NF-κB pathway, increased expression of adhesion molecules such as *ICAM1* and chemokine synthesis, such as *CXCL-1, -2* and *-5*. This activation of tumor cells appears to entail an accumulation of macrophages and in consequence also osteoclasts in the bone marrow [48]. Indeed, while osteoclasts and macrophages were not increased in the bone marrow of HFD-treated mice [28], they were strongly induced after melanoma challenge in HFD-treated mice. During the development of osteolytic bone metastases, osteoclasts and macrophages are essential for enhancing bone tumor growth and bone metastasis [49, 50]. Mechanistically, osteopontin appears to be important as it is released by macrophages and induce osteoclastogenesis [31, 51, 52]. Our analysis showed that osteopontin level was increased in HFD treated mice challenged with melanoma cells. Furthermore, deletion of osteopontin resulted in lower osteoclast numbers, suggesting that this cytokine is a key driver of melanoma-induced bone degradation. Surprisingly, osteopontin and IL-6 formed a positive loop, which led to CXCLs production from melanoma cancer cells. Also, osteopontin appears to act in a feed-back loop with cancer cells, stimulating their proliferation, as well as adipocytes, where osteopontin stimulates the synthesis of inflammatory cytokines such as IL-6 [53] and TNFα [54].

In conclusion, these findings provide a novel insight of how metabolic stress influences cancer. Fat accumulation in the bone marrow provides a niche for tumor cells promoting their proliferation by IL-6 and JAK2. In addition, the interaction of cancer cells with the bone marrow fat facilitates the accumulation of mononuclear cells and osteoclasts in the bone marrow by expressing C-X-C chemokines and osteopontin. Hence, the IL-6-JAK2- osteopontin axis is a key pathway for setting up the metabolic tumor niche and an interesting therapeutic target.

## MATERIALS AND METHODS

### Mice and treatment

Male C57BL/6N mice (6 week-old) were purchased from Jackson Labs and maintained at 25°C with 12-h light and dark cycles. OPN deficient mice were purchased from Jackson Labs (B6.128S6(Cg)-Spp1^tm1Blh^/J). Mice were fed with normal diet (ND; sniff, Cat.#D59494) or high fat diet (HFD; Research Diets, Cat.#D12330) ad libitum for 6 weeks. Intratibial injections of B16F10 cells were performed as previously described by Bakewell *et al*. [50]. Briefly, mice were anaesthetized with isoflurane (Abbott; IsoFlo®, Cat.#05260-05). For intratibial (i.t.) injection, 1×10^4^ B16F10 melanoma cells suspended in 50μl PBS or only PBS (vehicle) were injected into the tibiae of anaesthetized mice. The 27G^¾^ needle (BD, Cat.#03086999) was inserted into the mouse tibia for delivering the cells into the metaphysis. For blocking IL-6, 0.5 mg of IL-6 monoclonal antibody (BioXCell, Inc. Cat.#BE0046) was injected intraperitoneally (i.p.) every other day after the injection of B16F10 cells into the tibia (Figure 5C). For Janus kinase 2 (Jak2) inhibition, 17μg/kg of the Jak2 inhibitor AG490 (AdooQBioScience Cat.#A10047) was daily injected i.p. after the administration of B16F10 cells into the tibia (Figure 6A). All mice were sacrificed at the indicated time point after B16F10 cell inoculation.

### Cell culture experiments

Murine melanoma cell lines (B16F10) were obtained from the American Type Culture Collection (ATCC), and were maintained in alpha minimum essential medium (α-MEM, Invitrogen™, Cat.#32561) containing 100 ml/L of fetal bovine serum (FBS, Biochrom, Inc. Cat.#s0113) with 100 IU/ml penicillin and 10 μg/ml streptomycin (Gibco™, Cat.# 15140) at 37°C (5% CO_2_, 95% air). B16F10-cells were starved in serum-free α-MEM medium containing 1% antibiotics and antimycotics for 48 h. Then, 5×10^5^ cells/well or 5×10^4^ cells/well were plated into 6-well plate or 12-well plate, respectively and stimulated with medium containing 2% serum from normal diet (ND) or high fat diet (HFD) treated mice. For adipocyte/B16F10 co-cultures, bone marrow mesenchymal stem cells were prepared as previously described by Soleimani and colleagues [55]. Mesenchymal stem cells or the murine adipocyte cell line (14F1.1 cells, provided by Prof. Dov Zipori of the Weizmann Institute of Science, Rehovot, Israel) were differentiated into adipocytes by the addition of the adipogenic cocktail (5 μg/ml insulin, Sigma, Cat.#I2643; 0.5 mM 3-isobutyl-1-methylxanthine, Sigma, Cat.#I7018; 1 μM dexamethasone, Sigma, Cat.#D4902; and 2 μM rosiglitazone, Sigma, Cat.# R2408) for 2 days followed by stimulation with 5μg/ml insulin for additional 7 days. Adipocytes and B16F10 cells were co-cultured in 24-well Transwell inserts with a 4 μm pore size within Dulbecco's Modified Eagle Medium (DMEM, Invitrogen™, Cat.#10566). After 48 h of co-culture, adipocytes and B16F10 cells were separately harvested for RNA isolation and gene expression profile analysis.

### Migration assay

B16F10 cells were seeded in 24-well plates and pre-cultured with 2% FBS of α-MEM medium overnight. After scratched, the cell monolayer was gently rinsed with serum-free medium to remove the detached cells, and then treated for 8h with 2% serum isolated from normal diet (ND) or high fat diet (HFD) mice. Pictures were taken with phase contrast microscope and 10 × magnifications for multiple views of the scratched monolayer. Coverage rate was calculated as wound width covered (%) by comparing with initial scratch size (0 h) using ImageJ software [56].

### Bone histology and histomorphometry

Mouse femurs and tibias were fixed in 4% formalin and decalcified in 14% EDTA until the bone is flexible. Long bones were embedded in paraffin and sliced at equivalent coronal sections through the center of the bone with 1-2 μm thickness. Histological sections were stained with hematoxylin and eosin (H&E) and tartrate-resistant acid phosphatase (TRAP) activity. For the detection of TRAP positive cells in bones, 2 μm sections were proceed according the manufacturer protocol (Sigma 387A). Trabecular bone area was measured according to standard protocol [57] with the Osteomeasure Analysis System (OsteoMetrics, Decatur, Georgia, USA). Tumor area was assessed in hematoxylin & eosin stained sections by OsteoMeasure according to previously reported protocol [28, 58, 59]. Briefly, tissue sections were investigated at 4 × magnifications using Zeiss Axioskop 2 microscope (Zeiss, Inc. Marburg, Germany) directly below the growth plate at the distal end of the tibia or at the bone tumor area.

### Immunostaining

De-paraffinized, ethanol-rehydrated tissue sections were stained with monoclonal antibodies (mAb) against surface markers of melanoma (HMB45; Pierce™, Cat.#MA1-34759; dilution 1:50), IL-6 (BioXCell, Inc. Cat.#BE0046; dilution 1:100), OPN (R&D system, Inc.Cat.#AF808; dilution 1:500), and Ki-67 (Pierce™, Cat.#PA5-19462; dilution 1:50). Pre-treatment and revelation procedure were performed following the instruction of VECTASTAIN ABC Kit (Vector Laboratories, Inc. Cat.#PK-4001, 4002, and 4005). For fluorescence immunostaining, slides were treated with 0.2% Triton X-100 and blocked with 5% normal serum control after antigen retrieval with citrate buffer (10mM Citric Acid, 0.05% Tween 20, pH 6.0), and incubated with the primary antibody and then secondary antibodies. Slides were covered using a DAPI mounting medium (Vector Laboratories Inc. Cat.#H-1200). Images were acquired using a Nikon Eclipse 80i microscope, equipped with Sony DXC-390P digital camera and NIS-Elements BR2.2 software.

### Quantitative RT-PCR

Frozen long bones or cell lysates were homogenized with a Precellys (PeQlab, Inc. Erlangen, Germany) in peqGOLD RNAPure^TM^ reagent (PeQlab, Inc. Cat.#30-1030). mRNA was isolated according to the manufacturer's instructions, digested with DNaseI and reversely transcribed into cDNA using High-Capacity RNA-to-cDNA™ Kit (Thermo Fisher Scientific, Inc. Cat.#4368813). Quantitative real-time PCR was performed using SYBR Green I-dTTP (Eurogentec, Inc. Cat.#95054) on CFX96 Touch™ Real-Time PCR Detection System (Bio-Rad, Inc. California, USA). Samples were analyzed in triplicates. *HPRT* and *β-actin* were used as housekeeping genes. Primer sequences are listed in Table 1.

### *In vitro* osteoclast differentiation assay

For primary osteoclast assays, bone marrow (BM) cells from C57BL/6 mice (6-8 weeks) were incubated at 37°C in (100mm dish) in basal culture medium containing 5 ng/ml macrophage colony-stimulating factor (M-CSF) overnight (5% CO_2_, 95% air). 5×10^5^/well (24-well plate) of non-adherent cells were cultured in α-MEM supplemented with 5 ng/ml receptor activator of nuclear factor (NF)-κB ligand (RANKL) (PeproTech, Inc. Cat.#315-11) and 20 ng/ml M-CSF (PeproTech, Inc. Cat.#315-02). Medium was replaced every 3 days. Cells were cultured at 37°C for 5-7 days. Mature osteoclasts were either stained for TRAP, a marker of osteoclasts, using a Sigma TRAP kit (Sigma-Aldrich, Inc. Cat.#387) or harvested for RNA isolation. TRAP+ multi-nucleated (n ≥ 3) cells were considered as osteoclasts. The procedure for tumour cell/osteoclast co-culture was adapted from Yagiz and colleagues [60]. Briefly, 5×10^4^ B16F10 cells were pre-incubated with 2 % serum from ND or HFD mice at 37°C for 12h. The cells were then fixed with 2.5% glutaraldehyde for 1 min and immediately quenched with 1.5% glycine in PBS for 20 minutes. Fixed cells were washed and incubated overnight in complete medium before the co-culture with monocytes. The cells were cultured in α-MEM medium containing 5 ng/ml RANKL and 20 ng/ml M-CSF. Medium was replaced every 3 days. TRAP staining and TRAP+ quantification were performed.

### Flow cytometry analysis

Bone marrow (BM) cells were isolated by flushing long bones with PBS and filtering through a 70-μm cell strainer (BD, Inc. Cat.#352350). After red blood cell lysis, BM cells were analysed using PE-conjugated CD11b (BD, Inc. Cat.#553311), FITC-conjugated Gr-1 (eBioscience, Inc. Cat.#RB6-8C5) and APC-conjugated Ly6C (BD, Inc. Cat.#557661) antibodies with Gallios flow cytometer (Beckman Coulter, Inc. Brea, CA, USA).

### Multiplex cytokines and enzyme-linked immunosorbent assay (ELISA)

Chemokines and cytokines were measured using Mouse Th1/Th2 10plex FlowCytomix Multiplex kit (eBioscience, Inc. Cat.#BMS820FF) according to the manufacturer's instruction. Serum levels of IL-6 (R&D system, Inc. Cat.#DY406), osteopontin (R&D system, Inc. Cat.#DY441), leptin (R&D system, Inc. Cat.#DY498), and CXCL-1 (R&D system, Inc. Cat.#DY453) were measured using DuoSet ELISA Development kits according to the manufactured recommendations.

### Western blot analysis

Electrophoresis of 15 mg protein was performed. Following protein transfer into 0.45 μM nitrocellulose (NC) membrane (BD, Cat.#1620115), membranes were blocked with 5% skim-milk in TBS-T buffer (0.05%) for 2 h. Then, membranes that washed and were probed using the following antibodies: Nuclear factor kappa B (NF-κB) p65 (Cell signaling Technology, Inc. Cat.#8242; 1:500 dilutions), phospho-NF-κB p65 (Ser468) (Cell signaling Technology, Inc. Cat.#3039; 1:500 dilutions) and anti–β-actin mouse mAb (Sigma-Aldrich, Inc. Cat.#A5316; 1:5,000 dilutions), and the secondary antibodies. Chemiluminescent reagent (GE Healthcare, Cat.#RPN2106) and detected on Amersham Hyperfilm ECL (GE Healthcare, Cat.#28906839) were used for the revelation of the western blot.

### Statistics

All data are presented as mean ± SEM. The statistical significance was determined by student's *t* test or one-way ANOVA using GraphPad Prism software 5.0 (*p<0.05, **p<0.01, ***p<0.001).

## SUPPLEMENTARY FIGURES


