# Supplementary material for: High fat diet increases melanoma cell growth in the bone marrow by inducing osteopontin and interleukin 6
Source: Oncotarget. 2016 Mar 30;7(18):26653–69. doi: 10.18632/oncotarget.8474 (PMC5042005; doi:10.18632/oncotarget.8474)
Supplement: Supplementary file 1 [file oncotarget-07-26653-s001.pdf]

High fat diet increases melanoma cell growth in the bone marrow by inducing osteopontin and interleukin 6

SUPPLEMENTARY FIGURES

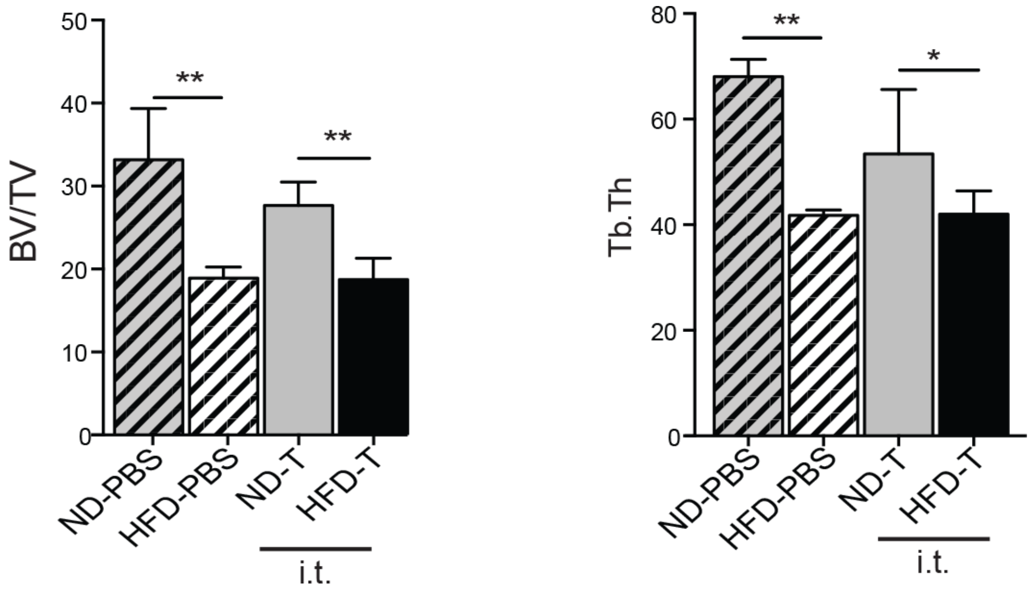

Supplementary Figure S1: Decreased bone volume in high fat diet mice. Bone volume and trabecular thickness in the tibiae of ND and HFD mice after i.t. B16F10 cells or PBS injection. All data are means ± SEM; n=6 to 8 per group. *p<0.05, **p<0.01, ***p<0.001.

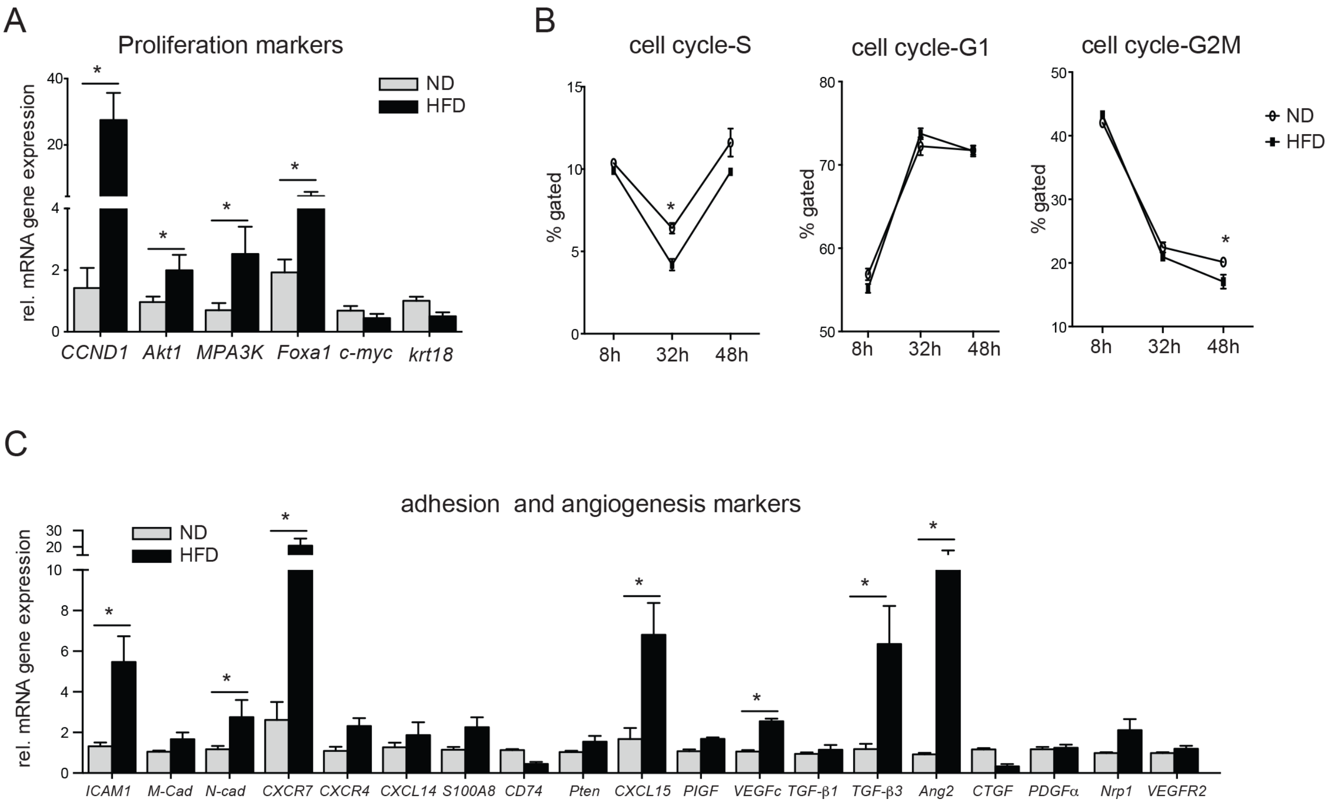

Supplementary Figure S2: B16F10 cells analyses after 2% high fat diet serum stimulation. A. Real-time analyses of proliferation marker genes in B16F10 cells after stimulation with 2% serum isolated from normal diet (ND) or high fat diet (HFD) mice. **B.** Cell cycle status analyses by FACS with anti-Ki-67 antibody and DAPI in B16F10 pre-incubated with 2% ND or HFD serum. **C.** Adhesion and angiogenesis marker genes expression in B16F10 cells after serum stimulation. All data are means ± SEM; 3 independent experiments were carried out in triplicate. *p<0.05, **p<0.01, ***p<0.001.

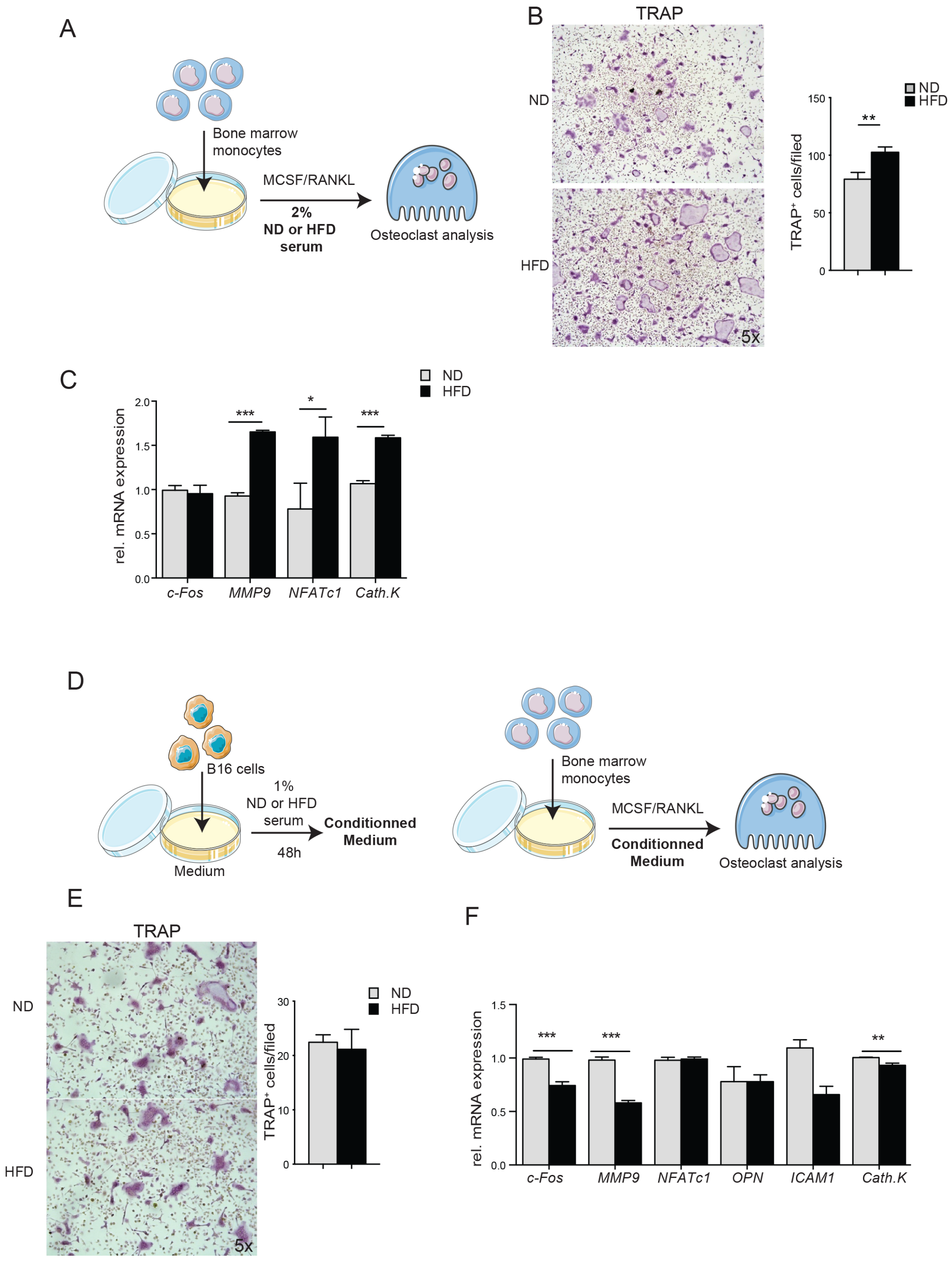

Supplementary Figure S3: Systemic factors from high fat diet mice induce osteoclastogenesis. A. Experimental setting: Bone marrow (BM) derived monocytes were treated with 2% serum isolated from normal diet (ND) or high fat diet (HFD) mice. **B.** TRAP staining pictures (magnification 5x) and TRAP positive cell quantification. **C.** Osteoclast marker genes expression after serum treatment. **D.** Overview of experimental design: B16F10 cells are coated on 100mm dish and stimulated for 48h with 2% serum isolated from ND or HFD mice. The conditioned medium (CM) is collected after starving for 24h. 50% CM is used to stimulate BM derived monocytes following a standard protocol for osteoclastogenesis. **E.** Representative pictures of TRAP staining (magnification 5x) and TRAP positive cells quantification. **F.** Gene expression of osteoclast markers in culture cells. All data are means ± SEM; 3 independent experiments were carried out in triplicate. *$p<0.05$.
